# Comparison of urine dipstick and albumin:creatinine ratio for chronic kidney disease screening: A population-based study

**DOI:** 10.1371/journal.pone.0171106

**Published:** 2017-02-02

**Authors:** Ji In Park, Hyunjeong Baek, Bo Ra Kim, Hae Hyuk Jung

**Affiliations:** Department of Medicine, Kangwon National University Hospital, Kangwon National University School of Medicine, Chuncheon, Gangwon-do, South Korea; Loyola University Chicago, UNITED STATES

## Abstract

Chronic kidney disease (CKD) is usually diagnosed using the estimated glomerular filtration rate (eGFR) or kidney damage markers. The urine dipstick test is a widely used screening tool for albuminuria, a CKD marker. Although the urine albumin:creatinine ratio (ACR) has advantages over the dipstick test in sensitivity and quantification of levels, the two methods have not been compared in the general population. A total of 20,759 adults with urinalysis data in the Korea National Health and Nutrition Examination Survey 2011–2014 were examined. CKD risk categories were created using a combination of eGFR and albuminuria. Albuminuria was defined using an ACR cutoff of 30 mg/g or 300 mg/g and a urine dipstick cutoff of trace or 1+. The EQ-5D index was used for the health outcome. Prevalence estimates of ACR ≥30 mg/g and >300 mg/g vs dipstick ≥trace and ≥1+ in adults aged ≥20 years were 7.2% and 0.9% vs 9.1% and 1.2%, respectively. For ACR ≥30 mg/g detection, the sensitivity, specificity, and positive/negative predictive values of dipstick ≥trace were 43.6%, 93.6%, 34.6%, and 95.5%, respectively. When risk categories created based on dipstick cutoffs were compared with those based on ACR cutoffs, 10.4% of the total population was reclassified to different risk categories, with only 3.9% reclassified to the same CKD category. Akaike information criterion values were lower, and non-fatal disease burdens of CKD were larger, in models predicting EQ-5D index using ACR-based categories compared to those using dipstick-based categories, even after adjusting for confounders. In conclusion, the urine dipstick test had poor sensitivity and high false-discovery rates for ACR ≥30 mg/g detection, and classified a large number of individuals into different CKD risk categories compared with ACR-based categories. Therefore, ACR assessments in CKD screening appear beneficial for a more accurate prediction of worse quality of life.

## Introduction

Chronic kidney disease (CKD) is a worldwide health problem related to premature death and poor quality of life. Management during its early stages may result in improved health outcomes [1]. Non-fatal health outcomes, including the health-related quality of life, are increasingly emphasized as an essential consideration in individual health care and population health planning. To quantify non-fatal disease burdens, the Global Burden of Disease Study 2010 measured the years lived with disability in a total of 291 diseases and injuries, and CKD was ranked as the 17th highest cause of years lived with disability in the United States [2]. CKD is defined as decreased kidney function or kidney damage that persists for more than 3 months. It is detectable with routine laboratory tests and is usually diagnosed with glomerular filtration rate (GFR) criteria or the presence of kidney damage markers. Although direct measurement of GFR is difficult, it can be estimated from the serum creatinine concentration. Albuminuria is the most frequently used marker of kidney damage in clinical practice and research.

Although the crude prevalence and characteristics of CKD can vary across the world [3–6], the global prevalence of early disease based on only albuminuria (i.e., CKD stages 1 and 2) is not less than that for advanced disease based on a decreased GFR (i.e., CKD stages 3–5). In a meta-analysis using worldwide population-based data, the age-standardized global prevalence of CKD stages 1–5 in adults aged ≥20 years in 2010 was reported to be 10.4% in men and 11.8% in women. Moreover, the prevalence of CKD stages 3–5 among adults aged ≥20 years was 4.7% in men and 5.8% in women [3]. This means that over 50% of CKD cases can be missed if albuminuria is ignored; thus, the measurement of albuminuria is inevitably important in the detection of CKD, particularly in the early stages.

Several studies have suggested that the urine dipstick test can be used to detect albuminuria in the general population [7,8]. Furthermore, the dipstick test is widely used as a low-cost screening tool. However, the urine albumin:creatinine ratio (ACR) has advantages in terms of sensitivity and quantification of levels; therefore, it is preferred to the dipstick in the current guidelines [9,10]. To date, a comparison in the utility of urine dipstick and ACR for CKD screening has not been evaluated in the general population.

The Korean National Health and Nutrition Examination Survey (KNHANES) allows for an evaluation of the usefulness of urine dipstick and ACR for CKD screening in the general population. This large, nationwide, representative survey conducted by the Korea Centers for Disease Control and Prevention (KCDC) includes laboratory examinations of urine dipstick, urine albumin, and urine/serum creatinine, allowing the identification and classification of CKD. Additionally, the survey included the EQ-5D health questionnaire to assess the health-related quality of life. The aim of this study was to compare the usefulness of the urine dipstick test and ACR for the diagnosis of CKD using KNHANES data as a population-representative sample.

## Materials and methods

### Subjects

The KNHANES is a nationwide cross-sectional survey on the health and nutritional status of non-institutionalized civilians in Korea. KNHANES comprises a health interview, health examination, and nutrition survey. KCDC conducted surveys in 1998, 2001, 2005, 2007–2009, 2010–2012, and 2013–2015. Written informed consent was obtained from each participant at enrollment. KNHANES was approved by the institutional review board of the Korea Centers for Disease Control and Prevention (IRB No. 2011-02CON-06-C, 2012-01EXP-01-2C, 2013-07CON-03-4C, and 2013-12EXP-03-5C).

**Fig 1:**
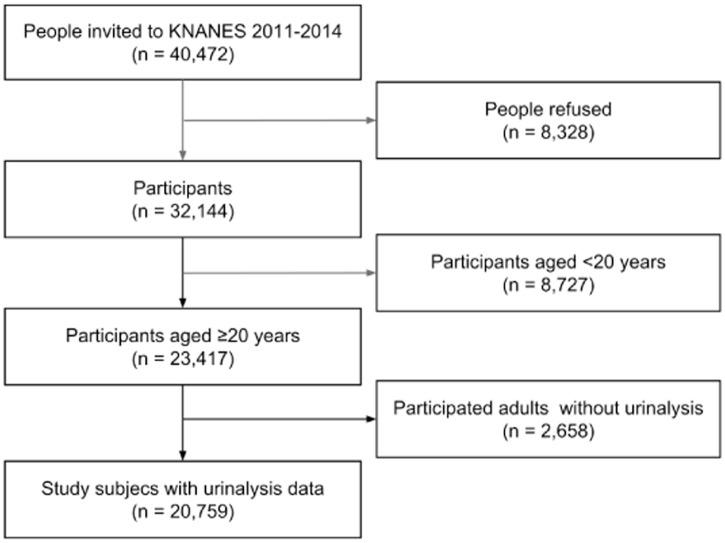
Flow chart of the study participant selection.

The present study was based on data from KNHANES 2011–2014 as urine albumin concentrations have only been measured since 2011, and data from KNHANES 2015 had not yet been released when this study was conducted. Among the 32,144 participants of KNHANES 2011–2014, we excluded participants younger than 20 years old (n = 8727) and adults without available urinalysis data (n = 2658). Following these exclusions, 20,759 subjects were included in this study (Fig 1).

### Health interview and health examination

As described in detail previously [11], the health interview and physical examination were performed by trained medical staff and interviewers at a mobile examination center. The health interview questionnaire, including the EQ-5D questionnaire, was collected via self-administration. Blood pressure was measured three times at 30 s intervals after a minimum of 5 min of rest in a seated position, and recorded as an average value of the second and third measurements. Blood samples were collected after at least 8 h of fasting (12 h, if possible), and random spot urine samples (first morning urine, if possible) were obtained from the subjects. The samples were properly processed, immediately refrigerated, and transported in cold storage to the central laboratory. Laboratory analyses were performed within 24 h after sampling. Dipstick urinalysis was performed using the Urisys 2400 cassette strip, which was read by a Urisys 2400 automated analyzer (Roche, Mannheim, Germany). Urinary albumin concentrations were measured using turbidimetric immunoassay with the Hitachi Automatic Analyzer 7600 (Hitachi, Tokyo, Japan). Serum and urine creatinine concentrations, standardized to isotope dilution mass spectrometry (IDMS), were measured using the colorimetric method with the Hitachi Automatic Analyzer 7600 (Hitachi, Tokyo, Japan) in KNHANES 2011 and 2012, and using the Jaffe rate-blanked and compensated method with the COBAS 8000 C702 (Roche, Mannheim, Germany) in KNHANES 2013 and 2014. Serum glucose concentrations were measured using an enzymatic method.

### Clinical characteristics

The GFR was estimated using the Chronic Kidney Disease Epidemiology Collaboration creatinine equation [12]. Spot urine ACR was computed in mg of albumin per g of creatinine (mg/g). We defined the reference standard of albuminuria as an ACR ≥30 mg/g or >300 mg/g, and used trace or 1+ as cutoff values for urine dipstick positivity. We ascertained kidney damage as an ACR ≥30 mg/g (or dipstick ≥ trace), and defined CKD as an ACR ≥30 mg/g (or dipstick ≥ trace) and/or an estimated GFR (eGFR) <60 mL/min/1.73 m^2^. In accordance with KDIGO guidelines, five eGFR categories (G1, eGFR ≥90 mL/min/1.73 m^2^; G2, eGFR 60–89 mL/min/1.73 m^2^; G3a, eGFR 45–59 mL/min/1.73 m^2^; G3b, eGFR 30–44 mL/min/1.73 m^2^; G4-5, eGFR <30 mL/min/1.73 m^2^), and three albuminuria categories (A1, ACR < 30 mg/g; A2, ACR 30–300 mg/g [or trace dipstick]; A3, ACR >300 mg/g [or dipstick ≥1+]) were employed in this study [9]. Since decreased eGFR and increased ACR are independently and additively associated with increased risks of adverse outcomes [13], the combination can multiply the risk of adverse outcomes. Risk categories estimating concurrent complications and future outcomes are considered helpful for the management of patients with CKD. Therefore, in subjects with CKD, the eGFR and albuminuria categories were classified into three risk categories according to the relative risk for adverse outcomes: moderately increased risk, G3aA1 or G1–2A2; high risk, G3bA1, G3aA2, or G1–2A3; and very high risk, G4–5A1, G3bA2, or G3a–5A3 [9].

Diabetes mellitus was defined by physician-diagnosed diabetes mellitus, a serum fasting glucose level ≥126 mg/dL, or the use of anti-diabetic drugs. Hypertension was defined by a systolic blood pressure ≥140 mmHg, diastolic blood pressure ≥90 mmHg, or the use of antihypertensive drugs.

Health-related quality of life was assessed using the Korean version of the EQ-5D questionnaire. The EQ-5D comprises 5 dimensions: mobility, self-care, usual activities, pain/discomfort, and anxiety/depression. Each dimension has 3 levels: no problem, some problem, and extreme problem. The EQ-5D index scores were calculated based on the responses to all dimensions. To score the EQ-5D index, we used the Korean value set, which has been established based on a representative national sample using the time—trade-off method [14]. Scores of 1 and 0 corresponded to optimal and worst health (judged to be equivalent to death), respectively.

### Statistical analysis

Statistical analyses were performed using SPSS (version 23.0; SPSS Inc., Chicago, IL, USA). The composite sample weight was introduced into our analyses to provide representative estimates of the Korean population. We calculated the composite weight by multiplying the survey weight by one-fourth for each of the years (2011–2014). The survey weight for subjects participating in the health interview and health examination was calculated using the sampling rate, response rate, and age/sex proportion of the Korean population.

Sensitivity, specificity, and positive and negative predictive values of the urine dipstick test were calculated using cross-tabulation tables for pairs of dipstick positivity ≥trace (or ≥1+) and the reference standard of an ACR ≥30 mg/g (or >300 mg/g). In addition to analyses for the total population, subgroup analyses according to age, sex, hypertension, and diabetes were conducted to identify potential effect modifications. We also cross-tabulated CKD risk categories (no CKD, moderately increased risk, high risk, and very high risk) pairing ACR-based categories vs dipstick-based categories, and calculated the proportion of each cell classified into the same or different categories.

Generalized linear models were used to predict the EQ-5D index as an outcome variable, using CKD risk categories as independent variables, with/without confounding variables including age, sex, hypertension, and diabetes. The group without CKD served as the reference CKD risk category. We used goodness of fit statistics to select a best-fitting model among different models for predicting EQ-5D index, and Akaike information criterion values were compared between candidate models. Additionally, we calculated CKD-specific, non-fatal burdens of disease as the prevalence of each CKD risk category multiplied by the adjusted mean difference of the EQ-5D index from the reference category, based on the approach of the Global Burden of Disease 2010 or WHO Global health estimates [15,16].

## Results

The weighted mean age of the study population was 46.6 years, and 52.0% were men (Table 1). The prevalence of the albuminuria category A2 with an ACR of 30–300 mg/g vs dipstick trace was estimated as 6.3% vs 7.9%, respectively, and for category A3 with an ACR >300 mg/g vs dipstick ≥1+ the prevalence was estimated as 0.9% vs 1.2%, respectively. The prevalence estimates for eGFR categories were as follows: G1, 68.3%; G2, 29.3%; G3A, 1.9%; G3B, 0.4%; and G4–5, 0.2%. The mean eGFR was 96.2 mL/min/1.73 m^2^ and decreased in subjects with albuminuria. Compared to subjects without albuminuria, those with albuminuria were older and had a higher prevalence of hypertension and diabetes.

**Table 1:** Demographic and clinical characteristics of the study population (study sample n = 20,759).

	ACR categories, mg/g			
	<30	30–300	>300	Total
Unweighted count, n	18,979	1,536	244	20,759
Population size, n	32,091,217	2,169,124	313,867	34,574,208
Age, years	45.8	57.1	58.4	46.6
Men, %	52.4	46.3	54.8	52.0
eGFR, mL/min/1.73 m^2^	97.0	87.8	72.4	96.2
Hypertension, %	23.7	57.3	77.9	26.3
Diabetes, %	7.6	29.3	45.8	9.2

Values are presented as weight-adjusted means or weighted percentage estimates.

ACR, albumin:creatinine ratio; eGFR, estimated glomerular filtration rate.

For the detection of albuminuria, the accuracy values of urine dipstick tests are as shown in Table 2. For the detection of ACR ≥30 mg/g, the sensitivity and specificity of the dipstick with a cutoff value of trace were 43.6% and 93.6%, respectively. Accordingly, the positive/negative likelihood ratios were 6.85 and 0.60, respectively, and the positive/negative predictive values were 34.6%, and 95.5%, respectively. While the sensitivity and specificity of dipstick tests were similar or slightly different between subgroups, the positive/negative predictive values of dipsticks varied widely. The positive predictive values for dipstick ≥trace for the detection of an ACR ≥30 mg/g were 26.6% and 20.9% in non-diabetics and non-hypertensives, respectively, compared to 62.4% and 58.1% in diabetics and hypertensives, respectively. The negative predictive values of dipsticks for detection of albuminuria were lower in participants with hypertension or diabetes compared to that in those without hypertension or diabetes. For the detection of an ACR >300 mg/g, the sensitivity and specificity of the dipstick test with a cutoff of 1+ were 75.4% and 99.5%, respectively. Accordingly, the positive/negative likelihood ratios were 157.93 and 0.25, respectively, and the positive/negative predictive values were 59.1%, and 99.8%, respectively.

**Table 2:** Diagnostic accuracy of urine dipstick results for detections of ACR ≥30 mg/g and ACR >300 mg/g (study sample n = 20,759).

	Dipstick ≥trace for detection of ACR ≥30 mg/g				Dipstick ≥1+ for detection of ACR >300 mg/g			
	Sensitivity	Specificity	PPV	NPV	Sensitivity	Specificity	PPV	NPV
Total population	43.6%	93.6%	34.6%	95.5%	75.4%	99.5%	59.1%	99.8%
Men	51.6%	92.5%	32.4%	96.5%	85.4%	99.4%	57.6%	99.9%
Women	36.4%	94.9%	38.0%	94.6%	63.3%	99.7%	61.9%	99.7%
20–39 years	57.5%	91.4%	17.3%	98.6%	96.9%	99.6%	34.3%	100.0%
40–59 years	46.5%	94.7%	37.8%	96.2%	78.6%	99.6%	62.6%	99.8%
≥ 60 years	36.6%	95.7%	61.2%	89.1%	68.2%	99.4%	69.6%	99.4%
No hypertension	43.7%	93.3%	20.9%	97.6%	78.2%	99.7%	38.7%	99.9%
Hypertension present	43.9%	93.8%	58.1%	89.6%	75.6%	99.1%	70.1%	99.3%
No diabetes	42.8%	93.6%	26.6%	96.8%	80.4%	99.7%	53.8%	99.9%
Diabetes present	47.9%	91.3%	62.4%	85.3%	73.6%	98.1%	62.7%	98.9%

Values are presented as weighted percentage estimates.

ACR, albumin:creatinine ratio; PPV, positive predictive value; NPV, negative predictive value.

The prevalence estimate of CKD defined as ACR ≥30 mg/g (vs dipstick ≥trace) or eGFR <60 mL/min/1.73 m2 was 8.4% (vs 10.8%). When we compared CKD risk categories created using the dipstick cutoffs of trace and 1+ with those using the ACR cutoffs of 30 mg/g and 300 mg/g, 10.4% of the total population were reclassified into different risk categories, with only 3.9% of the total population reclassified into the same CKD category (Table 3). Of the total population, 3.4% were reclassified from no CKD using dipstick-based categories to moderately increased risk using ACR-based categories, with 5.9% reclassified in the opposite direction. Approximately 0.9% were reclassified from moderately increased or high risk using dipstick-based categories to an increased risk using ACR-based categories, with 0.1% reclassified in the opposite direction.

**Table 3:** Reclassification across CKD risk categories based on the eGFR and ACR results from those based on the eGFR and dipstick results (analyzed n = 19,966).

		eGFR and ACR-based risk category				
eGFR and dipstick-based risk category		No CKD	Moderately increased risk	High risk	Very high risk	Total
No CKD	Population size	28,666,933	1,145,658	12,650		29,825,241
	(%)	(85.7%)	(3.4%)	(0.0%)		(89.2%)
Moderately increased risk	Population size	1,968,244	1,067,034	283,446	2,693	3,321,417
	(%)	(5.9%)	(3.2%)	(0.8%)	(0.0%)	(9.9%)
High risk	Population size		26,365	109,726	30,761	166,852
	(%)		(0.1%)	(0.3%)	(0.1%)	(0.5%)
Very high risk	Population size		775	10,920	125,524	137,219
	(%)		(0.0%)	(0.0%)	(0.4%)	(0.4%)
Total	Population size	30,635,178	2,239,832	416,741	158,978	33,450,729
	(%)	(91.6%)	(6.7%)	(1.2%)	(0.5%)	(100.0%)

Values are presented as weighted numbers or weighted percentage estimates.

CKD, chronic kidney disease; ACR, albumin:creatinine ratio.

**Fig 2:**
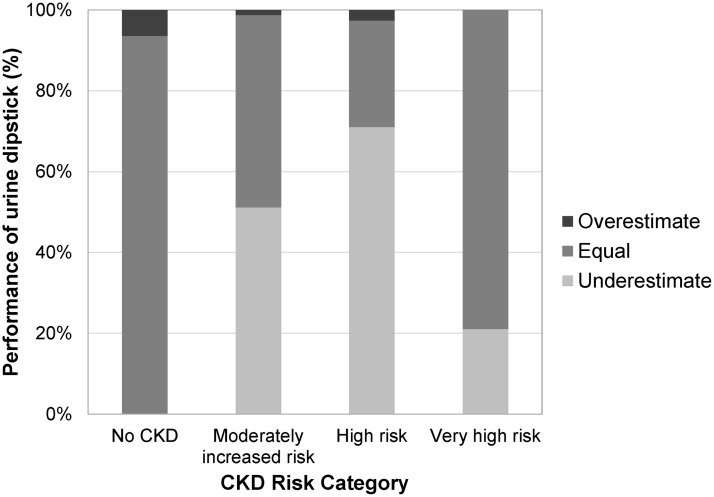
Performance of urine dipstick in estimating the CKD risk categories compared to albumin:creatinine ratio.

The reclassification rates for each risk category are shown in Fig 2. Over half of the subjects within the ACR-based moderately increased and high risk categories were classified into lower risk categories, when grouped based on the dipstick results.

**Fig 3:**
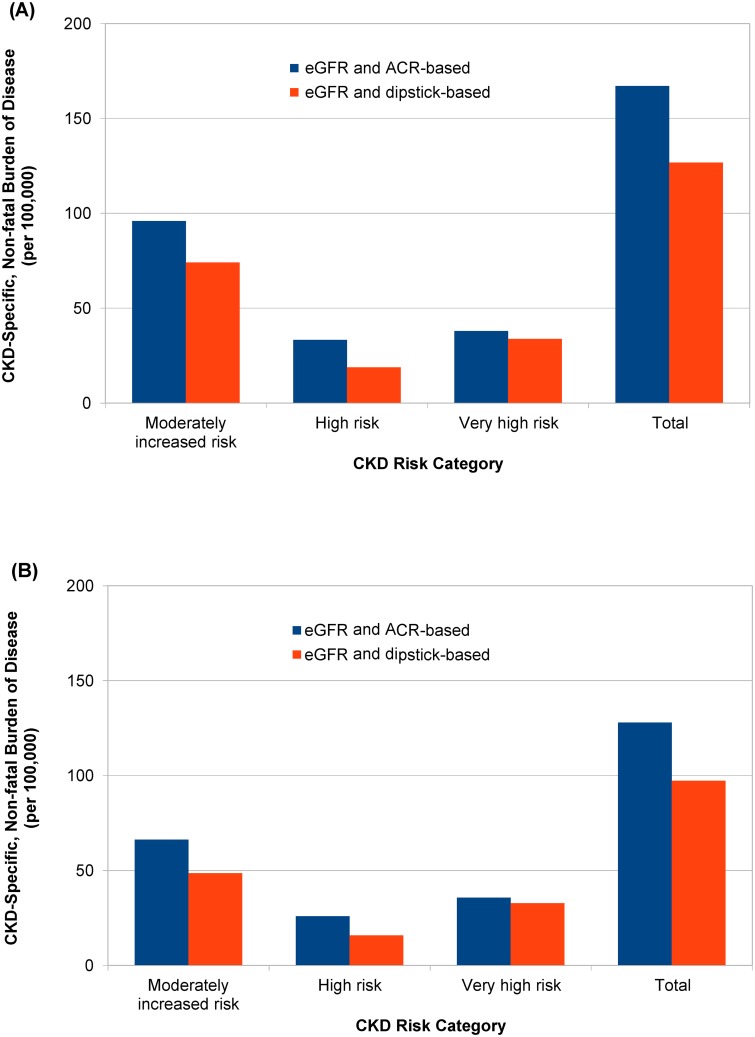
CKD-specific, non-fatal burden of disease (per 100,000 years). The values were calculated as the prevalence of each risk category multiplied by the mean difference of EQ-5D index from the reference without CKD: by the age and sex-adjusted difference (A) and by the age, sex, hypertension, and diabetes-adjusted difference (B).

Table 4 shows the models for predicting EQ-5D index by the CKD risk categories. In all models, the mean difference of the EQ-5D index from the reference (without CKD) increased progressively in higher risk categories. The models using the ACR and eGFR results had lower Akaike information criterion values compared to those using the dipstick and eGFR results, even after adjusting for confounders. Non-fatal disease burdens of CKD, which were calculated as the prevalence of each risk category multiplied by the mean difference of EQ-5D index from the reference, were larger in models using the ACR and eGFR results than in those using the dipstick and eGFR results (Fig 3A and 3B).

**Table 4:** Comparison of prediction for EQ-5D index between CKD risk categories based on the eGFR and ACR results and those based on the eGFR and dipstick results (analyzed n = 19,024).

			Model 1		Model 2		Model 3	
Risk categories of CKD	Population size	(%)	B^a^	(SE)	B^a^	(SE)	B^a^	(SE)
Based on eGFR and ACR								
No CKD	30,635,178	(91.6%)	0.000	-	0.000	-	0.000	-
Moderately increased risk	2,239,832	(6.7%)	-0.042	(0.003)	-0.014	(0.003)	-0.010	(0.003)
High risk	416,741	(1.2%)	-0.064	(0.007)	-0.027	(0.007)	-0.021	(0.007)
Very high risk	158,978	(0.5%)	-0.119	(0.011)	-0.080	(0.010)	-0.075	(0.011)
Akaike's Information Criterion			-27507.5		-29739.4		-29305.8	
Based on eGFR and dipstick								
No CKD	29,825,241	(89.2%)	0.000	-	0.000	-	0.000	-
Moderately increased risk	3,321,417	(9.9%)	-0.010	(0.003)	-0.007	(0.002)	-0.005	(0.002)
High risk	166,852	(0.5%)	-0.090	(0.011)	-0.038	(0.010)	-0.032	(0.011)
Very high risk	137,219	(0.4%)	-0.117	(0.012)	-0.082	(0.011)	-0.080	(0.012)
Akaike's Information Criterion			-27307.6		-29721.5		-29300.9	

Values are presented as weighted numbers or weighted percentage estimates. Model 1 was unadjusted. Model 2 was adjusted for age and sex. Model 3 was adjusted for age, sex, hypertension, and diabetes.

^a^ B = the mean difference of EQ-5D index from the reference category with no CKD.

CKD, chronic kidney disease; ACR, albumin:creatinine ratio; SE, standard error.

## Discussion

In this population-based study, urine dipstick tests had poor sensitivity and high false-discovery rates for the detection of an ACR ≥30 mg/g or CKD (although they were found to have a fair sensitivity and low false-omission rates for detection of an ACR >300 mg/g). When CKD risk categories created using eGFR and ACR results were compared with those created using eGFR and dipstick results, a large number of individuals were reclassified into different risk categories. Furthermore, the ACR-based CKD risk category was superior to the dipstick-based category in predicting worse health-related quality of life. These results suggest that urine dipstick test is an insufficient screening tool for the diagnosis of CKD, and that ACR is more beneficial in predicting the risk for a worse quality of life in the general population.

In our study of 20759 Korean adults aged ≥20 years, a dipstick ≥trace identified an ACR ≥30 mg/g with 43.6% sensitivity and 93.6% specificity, while a dipstick ≥1+ identified an ACR >300 mg/g with 75.4% sensitivity and 99.5% specificity. A previous study of 2321 Japanese adults aged ≥40 years showed similar results: 37.1% sensitivity and 97.3% specificity for a dipstick ≥trace in the detection of an ACR ≥30 mg/g, and 76.9% sensitivity and 96.5% specificity for a dipstick ≥1+ in the detection of an ACR >300 mg/g [7]. In another study of 11,247 Australian adults aged ≥25 years, the urine dipstick test had higher sensitivity but lower specificity than found in our study: a dipstick ≥trace identified an ACR ≥30 mg/g with 69.4% sensitivity and 86.8% specificity, and a dipstick ≥1+ identified an ACR >300 mg/g with 98.9% sensitivity and 92.6% specificity [8]. The differences in observed sensitivity and specificity may be explained by possible differences in calibration of the dipstick tests. In the Australian study, the prevalence rates for ACR ≥30 mg/g and ACR >300 mg/g were 6.6% and 0.8%, respectively, which were similar to ours (7.2% and 0.9%, respectively). However, the prevalence rates for a dipstick ≥trace and dipstick ≥1+ were 16.9% and 8.1%, respectively; which were much higher than ours (9.1% and 1.2%, respectively). Moreover, 72.9% of Australian participants with a dipstick ≥trace had an ACR of <30 mg/g (i.e., no albuminuria). If a dipstick ≥trace in the Australian study was used as an initial screening tool for albuminuria, 16.9% of the total population would require another visit for laboratory confirmation of their positive results, and 72.9% of the revisited candidates would be revealed to have no albuminuria. The urine dipstick test is a simple and inexpensive method, and can be easily repeated if the result of the initial screening is abnormal. However, the inconvenience of another visit for confirmatory testing can exceed the cost-effectiveness, according to circumstances.

In this study, the sensitivity and specificity of dipsticks were similar between the high-risk vs low-risk subgroups (elderly vs young subjects, diabetics vs non-diabetics, and hypertensives vs non-hypertensives). However, the positive predictive values varied widely between these subgroups (e.g., the values for a dipstick ≥trace for predicting an ACR ≥30 mg/g were in the 20% range in the low-risk subjects, compared to the 60% range in high-risk subjects). If urine dipstick was used for albuminuria screening in the low-risk subjects, about 80% of dipstick-positive results would turn out to be negative upon laboratory confirmation. In contrast, in high-risk subjects, the negative predictive values of a dipstick ≥trace for predicting an ACR ≥30 mg/g were between 85% and 90% (i.e., 10% to 15% of dipstick-negative subjects would have albuminuria with an ACR ≥30 mg/g).

We grouped CKD into three risk categories by the eGFR and albuminuria results. For comparison, we used two separate albuminuria criteria with dipstick cutoffs of trace and 1+ and with KDIGO-recommended ACR cutoffs of 30 mg/g and 300 mg/g because the prevalence rates of urine dipstick trace and dipstick ≥1+ were comparable to those of ACR 30–300 mg/g and ACR >300 mg/g, respectively. When we compared CKD risk categories created using the dipstick cutoffs to those using the ACR cutoffs, a large number subjects were reclassified into different risk categories, although the overall prevalence of CKD was comparable. If urine dipstick tests were used as an initial screening tool for CKD detection, 40.7% of CKD cases would be missed, and conversely 5.9% of the total population would be falsely diagnosed with CKD. Furthermore, an inaccurate prediction of the risk outcome in subjects with CKD may occur.

EQ-5D index scores, incorporating values for generic health states and the quality of life, were used for the outcome measure in the present study. EQ-5D has been widely used to measure health-related quality of life in various diseases and conditions [17], and has also been used in previous studies of patients with CKD [18,19]. The EQ-5D provides health utility scores as preference-based measures of health. Such utility scores facilitate the calculation of disability-adjusted life years, as used for the quantification of diseases burdens in the World Health Organization Global Health Estimates and World Bank-commissioned Global Burden of Disease study [20].

CKD is well known to be a risk factor for premature death [21,22] and a predictor of a worse quality of life [19,23,24]. In a meta-analysis of 10 cohorts with 266,975 subjects [13], eGFR and albuminuria were multiplicatively associated with all-cause and cardiovascular mortality; the findings in cohorts using dipstick data were comparable to those in cohorts measuring ACRs. A recent study of 3,446 Japanese subjects [25] showed that albuminuria based on an ACR ≥30 mg/g was more strongly associated with mortality, as compared to dipstick-positive albuminuria, suggesting that ACR measurements might be superior to dipstick tests in predicting poor prognosis. Additionally, to the best our knowledge, we demonstrated for the first time that CKD risk categories classified by ACR and eGFR results were superior to those created using dipstick and eGFR results in predicting the risk for worse health-related quality of life and in estimating CKD-specific, non-fatal burdens of disease. Therefore, we believe that ACR measurements are more beneficial than dipstick tests for CKD screening, in terms of predicting the risk for worse health-related quality of life, as well as premature death.

There are several limitations in the present study. First, the chronicity of kidney disease was not verified. Urine ACR and serum creatinine for each person were measured only once. Thus, transient albuminuria or acute kidney injury could not be excluded, and the prevalence of CKD could be overestimated. Future research evaluating the sensitivity and specificity of 1-time testing for CKD, defined as decreased kidney function or kidney damage persisting for at least 3 months, would help to clarify the usefulness of these tests. Second, we used ACR measured in a spot urine sample as the reference standard and did not measure albumin excretion rate in a timed urine sample. Although current guidelines recommend the use of spot urine testing for the calculation of ACR [9,10], there is no standardization for the collection and measurement of urine albumin. Third, our study used cross-sectional data and cannot elucidate the causal relationship between CKD risk categories and poor quality of life. Finally, there are insufficient studies evaluating the effectiveness of CKD screening. Our results suggest that CKD screening using ACR and eGFR is useful in the prediction of worse health-related quality of life. However, further research will be required to evaluate whether the identification and early treatment of CKD in individuals without even hypertension or diabetes can improve health outcomes, including the quality of life.

## Conclusions

The urine dipstick test had poor sensitivity and high false-discovery rates for the detection of an ACR ≥30 mg/g or CKD in the general population; however, it did possess fair sensitivity and low false-omission rates for the detection of an ACR >300 mg/g. A large number of individuals were reclassified into different CKD risk categories according to whether ACR or dipstick results were used for categorization. The screening and classification of CKD using ACR and eGFR results appear to be useful in accurately predicting the risk of worse health-related quality of life.
